# Integrating Pre-Exposure Prophylaxis Delivery in Public Health Family Planning Clinics: Lessons Learned From a Programmatic Implementation Project in Kenya

**DOI:** 10.3389/frph.2021.683415

**Published:** 2021-09-23

**Authors:** Kenneth K. Mugwanya, John Kinuthia

**Affiliations:** ^1^Department of Global Health, University of Washington, Seattle, WA, United States; ^2^Department of Epidemiology, University of Washington, Seattle, WA, United States; ^3^Kenyatta National Referral Hospital, Nairobi, Kenya

**Keywords:** family planning clinics, HIV prevention, implementation, pre-exposure prophylaxis (or PrEP), integrated services

## Abstract

Sexually active African women are a priority population for HIV prevention due to the disproportionately high frequency of new HIV infections. Family planning (FP) clinics offer an already trusted platform that can be used to reach women for HIV prevention services, including pre-exposure prophylaxis (PrEP). In the recent PrEP Implementation in Young Women and Adolescent (PrIYA program), we piloted PrEP implementation in FP clinics in Kisumu, Kenya, and demonstrated that it was possible to integrate PrEP provision in FP systems with a program-dedicated staff. In this perspective, we describe experiences and strategies employed to introduce PrEP implementation in FP clinics and lessons learned. We identified the following lessons for PrEP introduction in FP clinics in Kenya: (1) possible to integrate and generate high enthusiasm for PrEP delivery in FP clinics but persistence on PrEP is a challenge, (2) involvement of national and regional stakeholders is critical for buy-in, contextualization, and sustainability, (3) delivery models that do not integrate fully with existing staff and systems are less sustainable, (4) creatinine testing at PrEP initiation may not be necessary, (5) fully integrated HIV and FP data systems need to be developed, and (6) incorporating implementation science evaluation is important to understand and document effective implementation strategies. In summary, integration of HIV prevention and FP services provides an opportunity to promote one-stop women-centered care efficiently. However, a broader focus on delivery models that utilize existing staff and novel strategies to help women identify their own risk for HIV are needed to ensure greater success and sustainability.

## Introduction

In HIV high burden settings, many women concerned about avoiding or postponing pregnancy are also at elevated risk for HIV. A recent landmark clinical trial in eastern and southern Africa (the ECHO Trial), designed to evaluate the risk of acquiring HIV in HIV-negative women who used depot medroxyprogesterone acetate-intramuscular, the copper intrauterine device, or levonorgestrel found no substantial difference in the risk for acquiring HIV among women using any of the three common methods of contraception included in the study (1). However, the incidence of new HIV infections among the participants was very high, nearly 4%, with a higher rate among women under 25 years irrespective of the contraceptive method. These results have rightly spurred important discussions about the urgent need to strengthen the integration of reproductive health services with combination HIV prevention services, including pre-exposure prophylaxis (PrEP). PrEP as a recommended user-controlled strategy can play an important role in preventing HIV acquisition, especially for women. In many settings in Africa family planning (FP) clinics provide broad coverage for women in their reproductive years. In Kenya, 65% of sexually active unmarried women use a modern contraceptive and a substantial proportion (69%) access it through public health FP settings (2). Thus, integrating PrEP in FP clinics where women already trust providers could allow for one-stop comprehensive healthcare services for women. However, there is limited experience from real-world settings on approaches and strategies to best deliver PrEP in African public health settings. In the recent PrEP Implementation in Young Women and Adolescent (PrIYA) program (funded through PEPFAR DREAMS innovation challenge) (3, 4), we piloted the implementation of PrEP in FP clinics in Kisumu, Kenya, and demonstrated that it was possible to integrate PrEP provision in FP systems with program-dedicated staff (3). In this report, we describe how we approached the introduction of PrEP implementation in FP clinics and lessons learned to facilitate dissemination of these learnings in other low-income settings.

## Overview of the PrIYA Program

The project goal, methods, implementation, and primary quantitative results have been previously reported (3, 4). Briefly, PrIYA was a 2-year implementation project to reach adolescents and young women at high risk for HIV acquisition through integrated delivery of PrEP within routine maternal child health (MCH) and FP clinics in Kisumu, Kenya. PrIYA was part of the larger DREAMS Innovation Challenge funded by the President's Emergency Plan For AIDS Relief (PEPFAR). The overall goal of the project was to demonstrate the feasibility of integrating PrEP delivery in 16 public MCH and FP clinics. The project M & E Logic Model is provided in Supplementary Figure 1. The program was implemented between July 2017 and June 2018 as a collaborative effort between the University of Washington, the Kisumu County Department of Health, and 16 health facilities in Kisumu, Kenya. FP clinics at eight of the 16 facilities participated as a delivery point for PrEP. The implementation strategies to promote PrEP screening and provision in FP clinics included: training of existing health providers, stakeholder engagement, technical assistance, and demonstration of clinical PrEP provision by project-supported nurses embedded within FP clinics. Project-supported nurses performed only HIV risk counseling and provision of PrEP but did not participate in the delivery of FP services. At nearly all the eight clinics, women first completed other services including HIV testing and were then referred to a PrEP-dedicated nurse. Specifically, women of reproductive age accessing FP services were universally counseled by a PrEP program dedicated nurse for HIV behavioral risk factors and willingness to consider PrEP for HIV prevention. The screening was conducted according to the Kenya National Guidelines (5), guided by the Ministry of Health (MOH) risk assessment tool (RAST) that was used to initiate conversions with women about HIV risk and HIV prevention but not as a scoring tool for ruling in or out potential PrEP users. Kenya PrEP guidelines identified the presence of any of the following behavioral factors in the last 6 months as an indication for substantial ongoing risk of acquiring HIV include the following: inconsistent or no condom use; having a high-risk sex partner(s) and of unknown HIV status; engaging in transactional sex; history of ongoing intimate partner and gender-based violence; recent sexually transmitted infections self-reported or etiologically diagnosed; recurrent use of post exposure prophylaxis; recurrent sex under the influence of alcohol/recreational drugs; injecting drug with shared needles and/or syringes; and having an HIV positive partner.

## Process and Lessons Learned

Moving novel interventions to scaled implementation requires adaptations to better fit within complex contexts, needs of the local target population, or to respond to unanticipated challenges (6, 7). Due to restrictions on the use of funds on research-related activities, the PrIYA program did not embed rigorous implementation science research, including the application of qualitative interviews with health providers or individual women that would have provided important insights into the implementation process and relevant contextual factors for integrated delivery. Nonetheless, we used multiple sources to document and understand the process of integrating PrEP provision in FP clinics in Kenya. The sources included: abstraction of program data, technical assistant reports, debrief reports from clinical training and stakeholder engagement, and observations. In this narrative, to supplement our published quantitative analysis, we describe our experiences and lessons learned that are organized under 10 themes: (1) Data collection and systems; (2) Demand creation, initiation, and continuation; (3) Service delivery models; (4) Stakeholder engagement and facility preparation; (5) Training and capacity strengthening for PrEP implementation; (6) PrEP commodity supply chain; (7) PrEP laboratories; (8) New clients to FP clinics; (9) Consent for programmatic and research activities; and (10) Importance of integrating rigorous implementation science evaluation.

### Data Collection and Systems

In this perspective, we present how the program and clinical data were obtained. The lack of robust data systems to track clients longitudinally is a key challenge in many FP clinics in low-income settings. For the PrIYA project, we used MOH/NASCOP data collection tools for HIV risk assessment and initiation of clients, which included RAST tool to guide PrEP eligibility and a clinical encounter form (PrEP card) for those who initiated PrEP. Program data including demographics, behavioral-risk characteristics, reported partner HIV status, PrEP uptake, self-reported adherence to PrEP, and adverse events were abstracted daily by program nurses and entered daily into tablets for upload to a server. Continuation and adherence to PrEP were assessed by self-report and PrEP refill records at the clinic and through follow-up phone calls to ascertain PrEP continuation status and reasons for discontinuing PrEP. In addition, we used MOH daily activity registers and drug accountability registers. For project-specific activities, referral books were used to document and track clients referred for services to relevant departments, including referral for other gender-based violence and treatment of sexually transmitted infections. Of note, although service provision for PrEP and FP was integrated, data tools remained mostly vertical with RAST, PrEP card, and M & E registers for PrEP services completed separately from the FP register for FP services. This unintegrated data system meant that staff had to complete duplicate forms and registers. Thus, robust and fully integrated data systems to monitor PrEP clients that align with other databases in FP clinics need to be developed to streamline longitudinal tracking of clients. We found that simple CME-liked training of health providers on proper documentation and reporting procedures helped to streamline reporting of MOH/NASCOP-mandated PrEP indicators.

### Demand Creation, Initiation, and Continuation on PrEP

Methods and analysis for quantitative data from the project have been previously reported. Briefly, we found high enthusiasm for PrEP from women accessing FP clinics. Overall, 1,271 were screened for HIV risk of whom 42% were < 24 years (3). The majority of women who reported were using injectable (56%) or implant (31%) methods for contraception, with only 5% reporting to be on oral contraception pills. Although in a community online survey >87% of women had heard about PrEP and 75% clearly understood who could be eligible for PrEP (8), demand for PrEP was mostly provider-driven through counseling by healthcare providers and facility-based healthcare talk in the waiting areas. More than one-third of women did not know the HIV status of their male partners.

**Figure 1:**
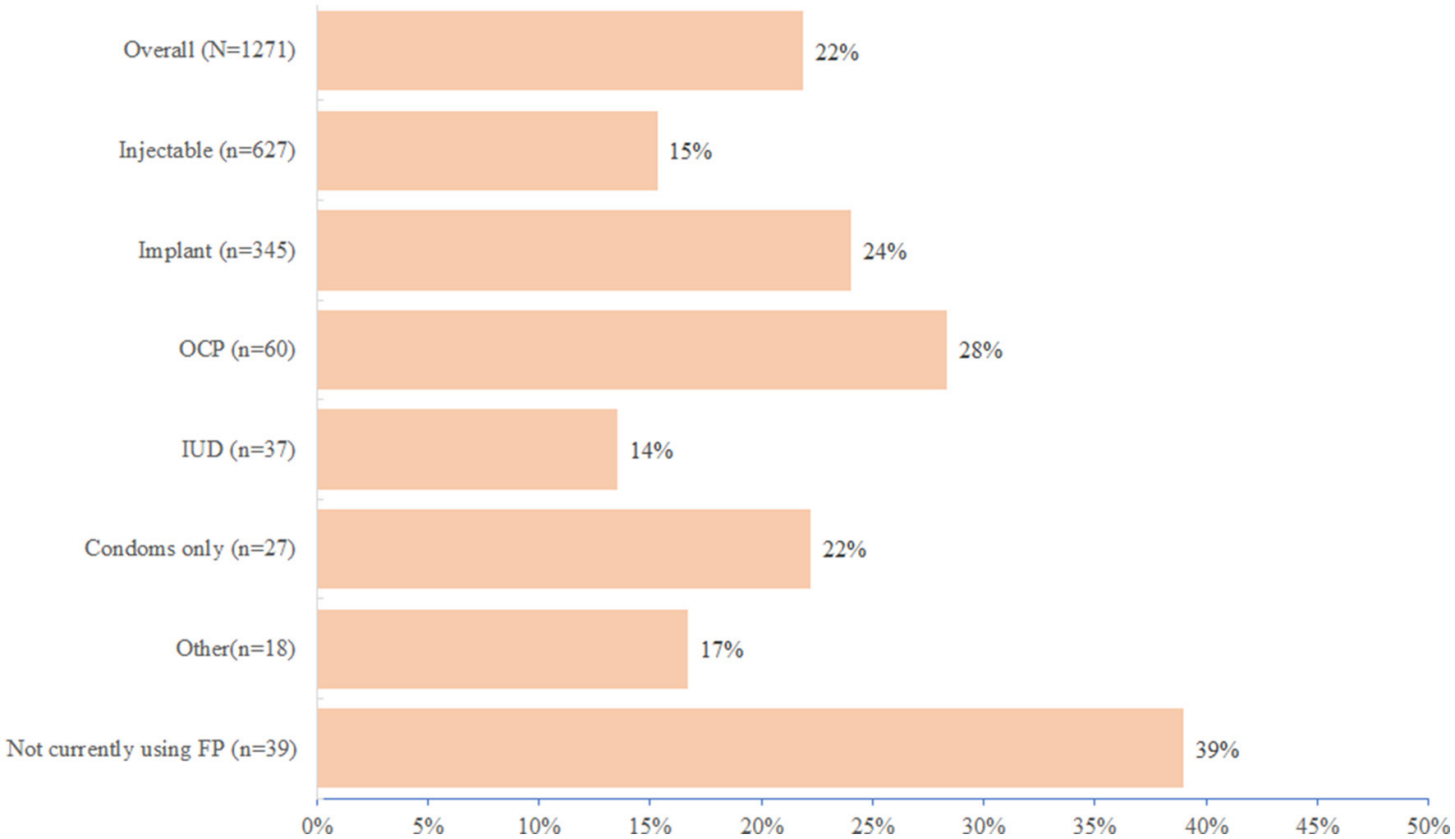
Frequency of PrEP initiation by contraception method used.

**Figure 2:**
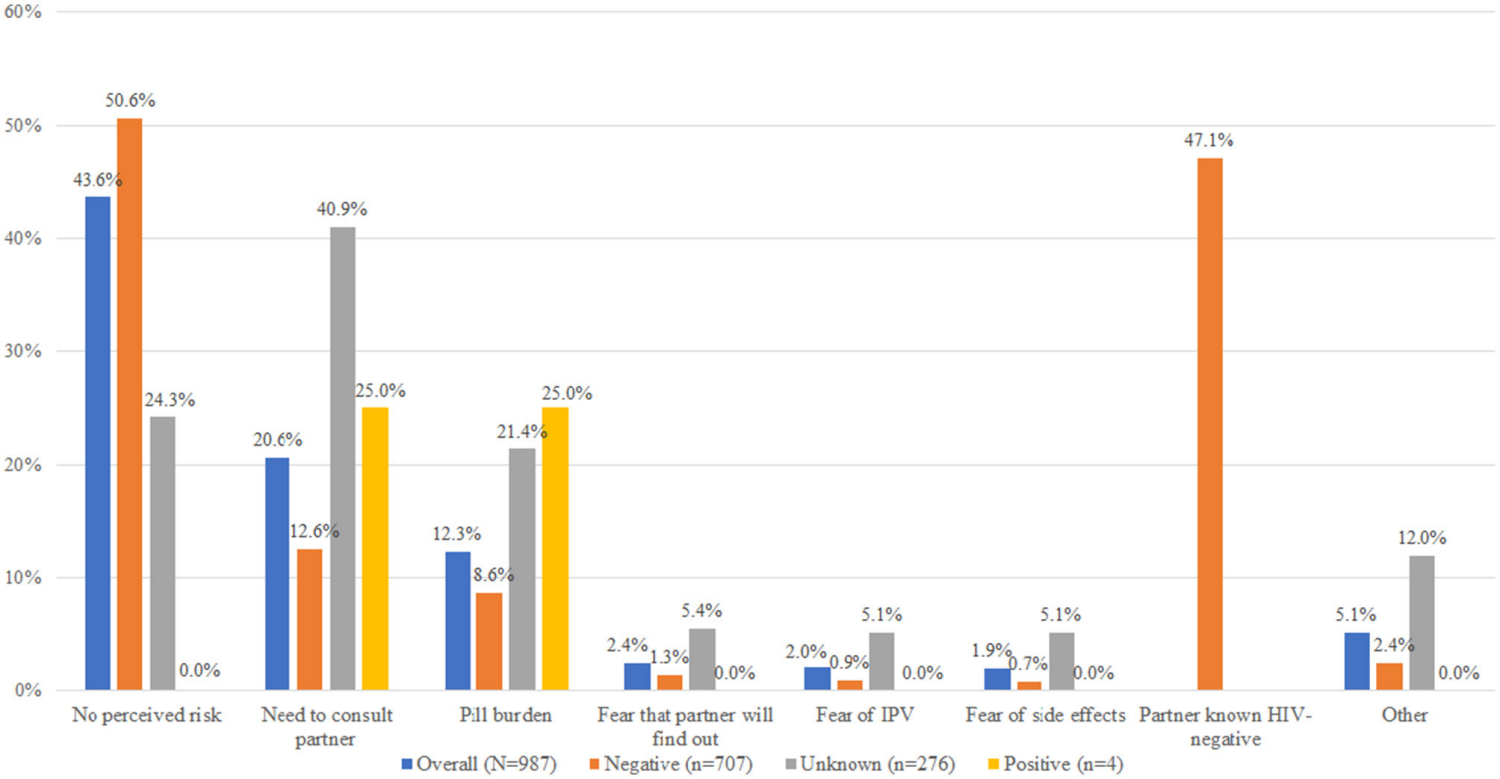
Reasons for declining PrEP by reported male partner HIV status.

Nearly 22% (278) of all women screened for HIV risk accepted PrEP with acceptance > 90% among women with at least one risk factor for HIV as per the Kenya PrEP guidelines (3). A higher proportion of women not on any contraception at the screening visit (39%) and those on the oral contraceptive pill (28%) initiated PrEP compared with only 24% of women on the implant and 15% on injectable contraception (Figure 1). Women ≥ 24 years more frequently elected to initiate PrEP compared to women < 24 years (69 vs. 31%) and were more likely to perceive or self-assess to be at risk for HIV than women < 24 years. Overall, among women screened and elected not to initiate PrEP (*n* = 987), 45% reported to have the low-perceived risk for HIV, and more than one-third (427/1,271) reported partners of unknown HIV status. We found that a substantial proportion of these women with partners of unknown HIV status (> 40%) still felt that they needed to consult their male partners before they could consider PrEP (Figure 2). This was a surprising observation given that PrEP as a user-controlled prevention option is expected to empower young at-risk women to have control of their own HIV prevention choices. Because we did not do any qualitative research, we were unable to gain important insights into this emergent theme. Of note, there were no important variations in reasons for no acceptance of PrEP by contraception method used (Supplementary Figure 2).

As observed in most PrEP studies in women, continuation was a challenge with sharp declines in use within months of initiation—and often within the first month. Overall, 41% returned for their first refill visit; there were variations by contraception method used: 56% for those not initially on any contraption method, 39% for injectable, 35% for oral contraception pill, 30% for an implant, and 20% for women IUD (Supplementary Figure 3). Awareness of PrEP and perceived risk of individuals for HIV were main drivers of continuation, with higher continuation rates observed among women who reported an HIV-positive male partner or those who were self-assessed to be at risk of acquiring HIV. Thus, in addition to advancing more prevention methods to provide more options and choices to address varying experiences and preferences of women, defining strategies that support women to better evaluate their own risk for HIV is equally important. In the program, we promoted male partner testing through distribution of HIV self-test kits that allowed some women with male partners of unknown HIV status to make informed decisions about their risk for HIV and needs for PrEP (9).

### Service Delivery Models

The primary strategy to integrate PrEP delivery in FP clinics was a program-supported nurse-led delivery of HIV risk counseling and provision of PrEP for women accessing routine FP services. New nurses hired specifically for the project and embedded into FP clinics were trained on screening and PrEP provision using a 2-day case-based interactive Kenya MOH PrEP curriculum. Project nurses only performed HIV risk counseling and provision of PrEP but did not participate in the delivery of FP services. Medically eligible women who wanted to initiate PrEP received same-day PrEP. As previously reported, across clinics, two main delivery services models for PrEP delivery were implemented: (1) codelivery where FP and PrEP services were delivered by the same FP nurse or (2) sequential services in which PrEP services by a PrEP-dedicated project nurse were offered after the client had completed their routine FP services (10). Common reasons for using a co-delivery approach instead of a sequential approach included not having a separate space allocated for PrEP services and having the high client to provider ratio, making it infeasible to allocate a nurse specifically for PrEP services. Screening for HIV risk was conducted according to the Kenya PrEP national guidelines (5), guided by a Kenya MOH RAST that was modified to include self-assessed reasons of women for choosing or declining PrEP. Provision of PrEP was subsequently transferred to the facility staff after projected funding had ended. Importantly, we found that PrEP uptake declined substantially when program-dedicated staff left after the project ended, demonstrating the need for PrEP delivery models that integrate fully with existing service delivery models and staffing.

### Stakeholder Engagement and Facility Preparation

Government support and program ownership is the key to the success and sustainability of African PrEP programs. PrEP roll-out nationally in Kenya was officially launched in May 2017, making it the first African national PrEP program and delivery and is slowly expanding (11). In the devolved government structure of Kenya, the MOH sets and guides national policy and guidelines, development of tools, and convenes implementation partners while County governments are responsible for service delivery. The PrIYA program worked closely with the Kisumu County government in planning the project and ensuring that PrEP medications were available at the healthcare facilities. The selection of clinics was conducted in consultation with the County-Government-based clinical volume and geographical location. The PrIYA project team was part of the Kenya national and County PrEP Technical Working Groups (TWG, which is charged with guiding PrEP implementation) but no direct financial support was provided to MOH or the County. Importantly, the project used the TWG platform to offer guidance and technical support needed to deliver PrEP in FP clinics on a national level.

### Training and Capacity Strengthening for PrEP Implementation

At the start of the project, 40 program-dedicated newly hired nurses were trained by the project leadership team on clinical PrEP delivery per national guidelines and subsequently deployed at the 16 MCH and FP clinics; eight nurses were deployed in FP clinics. Program nurses thereafter worked with the Kisumu County Health authorities to support the readiness of clinics to deliver PrEP in FP clinics in a combination HIV prevention package. At each of the implementing clinics, program nurses conducted sensitization sessions to introduce the program and seek advice on the best ways to integrate PrEP delivery at the facility. Six months prior to the end of the study project, we conducted facility-wide training (i.e., to train other providers beyond FP clinics). The purpose of this effort was to coach and mentor existing healthcare providers as a sustainability plan to transfer the provision of PrEP services from project-dedicated nurses after project funding had ended. Overall, a total of 554 existing MOH healthcare providers (an average of 34 per facility) from MCH and FP clinics were trained in competencies in the following domains: (1) HIV risk assessment, counseling on PrEP initiation, discontinuation, adherence, and interpretation of PrEP-related laboratory tests, (2) Sensitization of women about PrEP in FP and MCH clinics, (3) Standardized clinical tools for promoting engagement of their male partner for HIV testing, and (4) Interpretation and use of clinical-level data to monitor women on PrEP. Subsequently, at the request of the Kisumu County government, we expanded our training and mentorship to an additional 21 ministry of health facilities where we trained 160 health providers on PrEP delivery. At these clinics, mentorship training was separated into short didactic and practical modules covered over 3–5 days at the facility. We found that this modular training provided flexibility and an effective format to provide on-job PrEP training for healthcare workers in public healthcare facilities without requiring them to leave the facility or disrupting other service provisions.

### PrEP Commodity Supply Chain

Pre-exposure prophylaxis commodities, including PrEP medications and HIV testing kits, were provided from the national program and supplied by the Kenya Medical Supplies Agency at no cost to women. HIV uninfected women at substantial risk for HIV infection who chose to initiate PrEP received PrEP as part of the Kenya National PrEP Program. Prior to starting, program staff worked with clinic staff, county-level health officials, and the Kenya Medical Supplies Agency to make PrEP commodities available within FP clinics. Because the national PrEP program was in the startup phase, cases of commodity stockouts were frequent in the early phase (first 6 months) mostly resulting from under projection of the required quantities. When stock-outs of PrEP commodities occurred, the program worked closely with the County and Sub-County pharmacists to redistribute PrEP drugs from healthcare facilities with adequate stock to those who had stock-outs which ensured continuity of services at the affected clinics.

### PrEP Laboratories

The Kenya PrEP guidelines recommend creatinine testing at baseline to evaluate kidney function but advise that the absence of test results should not delay PrEP initiation (5). In the Health system of Kenya, the cost of most laboratory testing including creatinine is met by the user, and mandating creatinine before PrEP initiation has the potential to be a significant barrier to access to PrEP services. Previous studies of PrEP safety found the risk of suboptimal kidney function to be very rare and no more frequent among PrEP users compared to non-PrEP users (12–17). In a subsect of the utility of point-of-care (POC) creatinine testing at PrEP initiation nested within the PrIYA project (18), we found that implementation of POC creatinine testing was feasible and performed more conservatively than laboratory-based testing (Roche Cobas c111 Analyzer; Roche Diagnostics, Indianapolis, IN). Importantly, POC testing results were available in a median of 1 min at a cost of $4.5 per test compared to 3.5 h at $5 per test for the standard laboratory testing. We found that in our project population of young healthy women, PrEP ineligibility due to suboptimal kidney function was very rare (only 0.02%) (18), suggesting that not requiring creatinine testing at PrEP initiation will generally be a safe decision (18). For those who sustain PrEP use annual testing may be adequate.

### New Clients to FP Clinics

To advance comprehensive HIV prevention services, the PrIYA project actively promoted knowledge of male partners with partner invitation and secondary distribution of HIV self-test kits (results presented elsewhere) (9). We observed that offering PrEP services and promotion of male partner testing attracted new clients to FP and MCH clinics that included women who came to FP clinics solely for PrEP services and some male partners who responded to clinic-based partner testing invitations. FP settings are traditionally not set for male partners but as efforts to integrate comprehensive HIV prevention services in FP settings take effect, it is imperative that providers prepare to serve clients across the gender spectrum.

### Consent for Programmatic and Research Activities

An early phase of implementation of a new biomedical intervention like PrEP is associated with uncertainties about requirements for consenting and how to manage priority populations that may not have been included in efficacy clinical trials. For PrEP, important populations excluded from efficacy trials included individuals younger than 18 years, pregnant and lactating women. In this project, we learned that it is possible to work with oversight ethical authorities to define and separate research procedures from program activities. In the PrIYA project, research procedures were defined as those activities not required for the clinical provision of PrEP but are important to understand how the overall program works, for which written informed consent was required. Written informed consent was obtained for all research procedures, for example, dried blood spots for tenofovir levels to evaluate adherence to PrEP. For program procedures related to standard procedures for PrEP counseling and provision of (i.e., HIV testing and counseling, PrEP prescription, and dispensing), the local IRB determined them to be of minimal risk for which only oral consenting was obtained, which helped to overcome an important barrier to access PrEP in this population.

### Incorporating Implementation Science Evaluation Is Important to Understand Delivery

Moving novel interventions from research into real-world settings presents challenges on how to adapt and fit the novel intervention or practice into complex contexts. Incorporating implementation science studies is important to document and understand what works for whom and under what circumstances and determining the best strategies for successful implementation interventions in real-world settings. Restrictions on the use of funds for certain research activities prohibited the PrIYA program to conduct rigorous process evaluation research, including qualitative interviews that would have provided additional insights into the implementation process and relevant contextual factors. Despite the limitations, triangulation of multiple data sources permitted the project to document and learn important lessons to the extent possible about working in public health FP clinics in this setting.

## Conclusion

Ensuring that African young women have access to effective contraception and are also able to protect themselves from HIV is critical to optimize their health and ending the HIV epidemic. The PrIYA program pioneered the implementation of PrEP delivery in real-world African FP settings, demonstrating that it is feasible and practical to gain efficiencies with a one-stop station for HIV prevention and FP services using existing public health FP infrastructure. Because of the strong support of the Kenyan Ministry of Health for PrEP as an important HIV prevention intervention, Kenya is an incubator for research on innovative PrEP delivery models, and lessons learned have the potential to inform and guide the expansion of PrEP delivery in other African settings.

## Data Availability Statement

The raw data supporting the conclusions of this article will be made available by the authors, without undue reservation.

## Ethics Statement

The studies involving human participants were reviewed and approved by The Human Subjects Division of the University of Washington and the Kenyatta Nation Hospital Ethical Review Committee approved the project and approval was obtained from the Kisumu County administration and facility in charges. Written informed consent from the participants' legal guardian/next of kin was not required to participate in this study in accordance with the national legislation and the institutional requirements.

## Author Contributions

All authors listed have made a substantial, direct and intellectual contribution to the work, and approved it for publication.

## Funding

KM was supported by funding from the US National Institute of Health Grant # R00MH118134 and R01MH123267.

## Author Disclaimer

The parent PrIYA Program was funded by the United States Department of State as part of the DREAMS Innovation Challenge (Grant # 37188-1088 MOD01), managed by JSI Research & Training Institute, Inc. (JSI). The opinions, findings, and conclusions stated herein are those of the authors and do not necessarily reflect those of the United States Department of State or JSI.

## Conflict of Interest

The authors declare that the research was conducted in the absence of any commercial or financial relationships that could be construed as a potential conflict of interest.

## Publisher's Note

All claims expressed in this article are solely those of the authors and do not necessarily represent those of their affiliated organizations, or those of the publisher, the editors and the reviewers. Any product that may be evaluated in this article, or claim that may be made by its manufacturer, is not guaranteed or endorsed by the publisher.
