# Supplementary figures and images for: Integrating Pre-Exposure Prophylaxis Delivery in Public Health Family Planning Clinics: Lessons Learned From a Programmatic Implementation Project in Kenya
Source: Front Reprod Health. 2021 Sep 23;3:683415. doi: 10.3389/frph.2021.683415 (PMC9580668; doi:10.3389/frph.2021.683415)

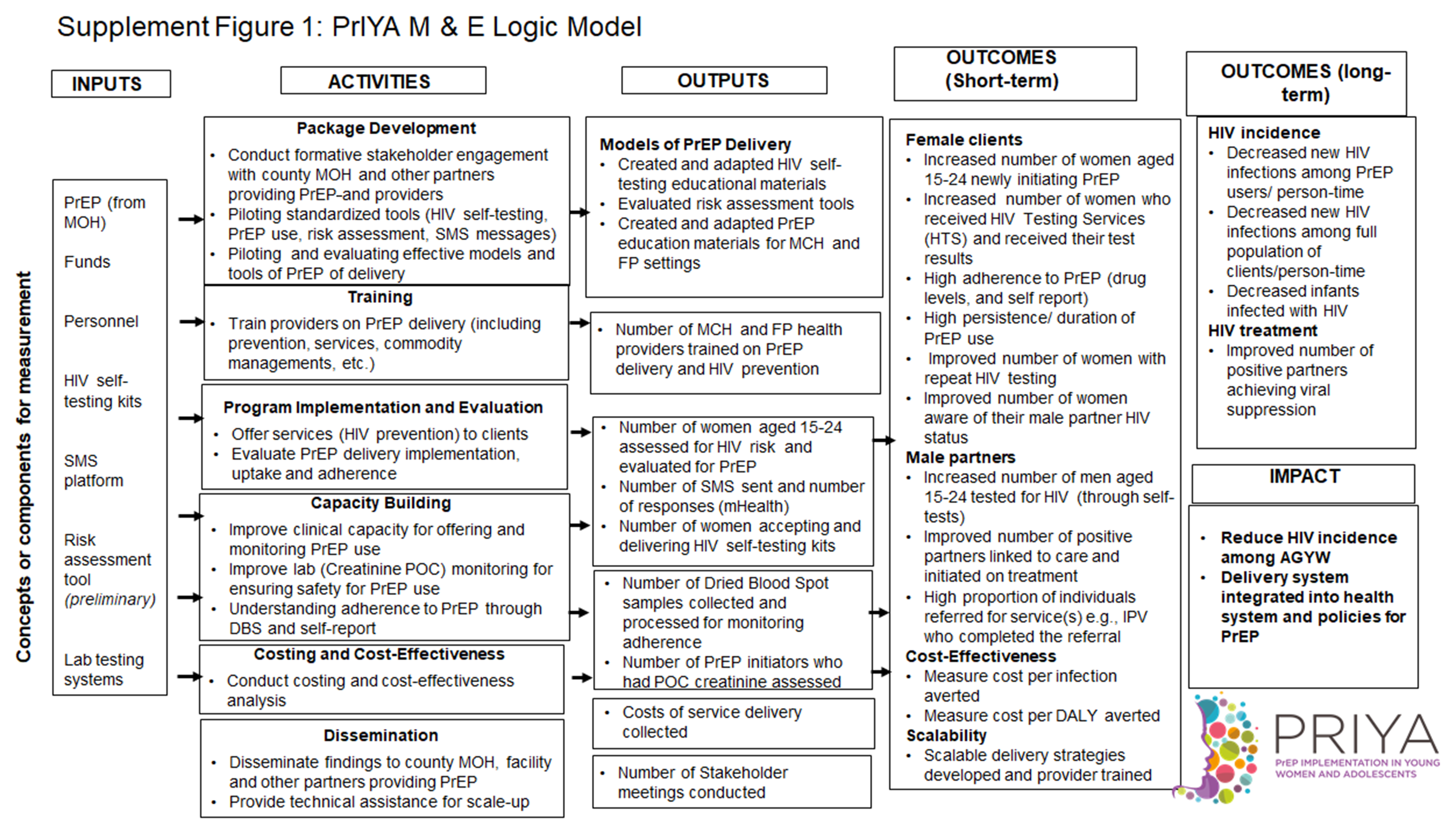

Supplement: Supplementary file 1 [file Image_1.TIF]

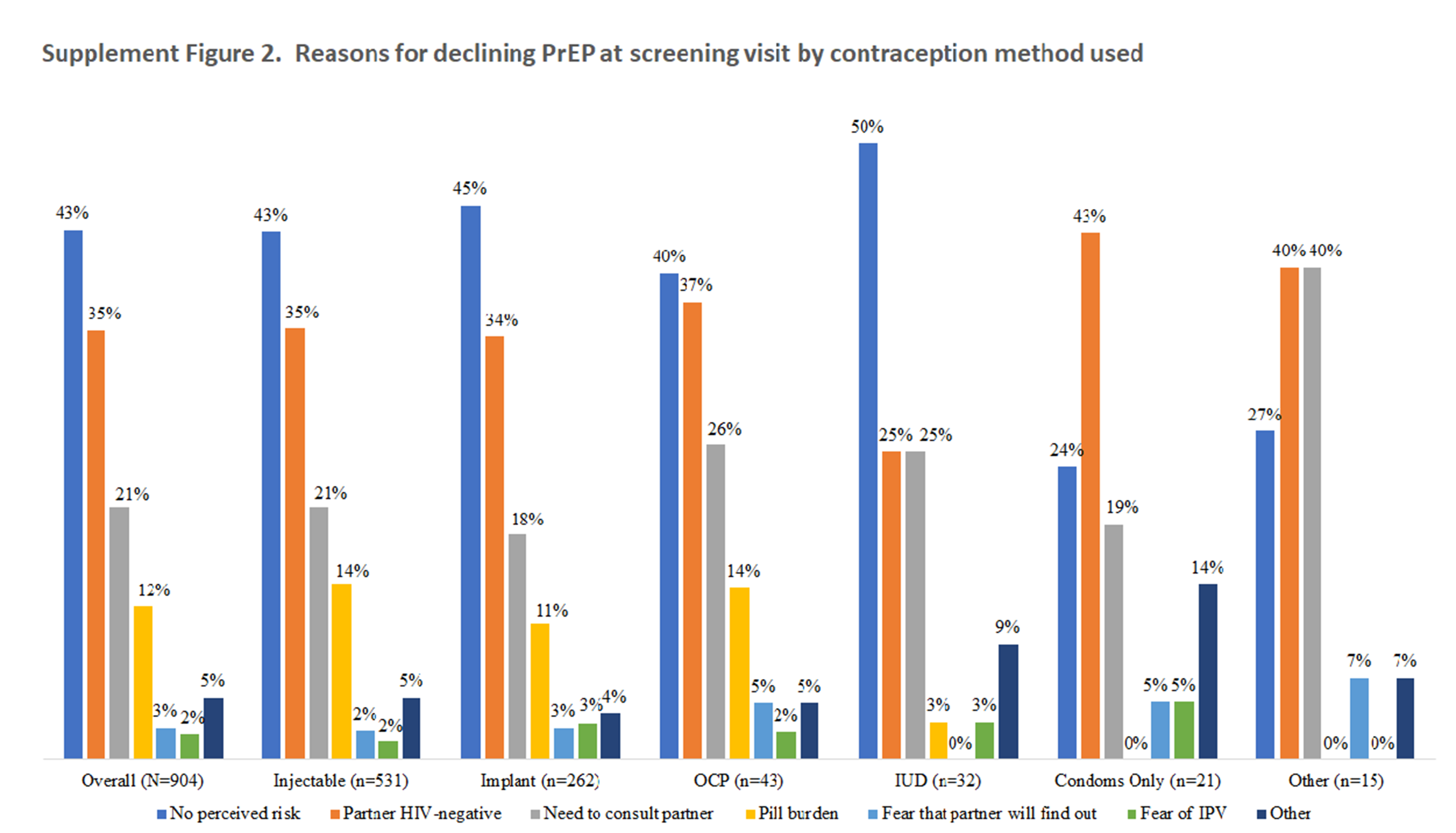

Supplement: Supplementary file 2 [file Image_2.TIF]

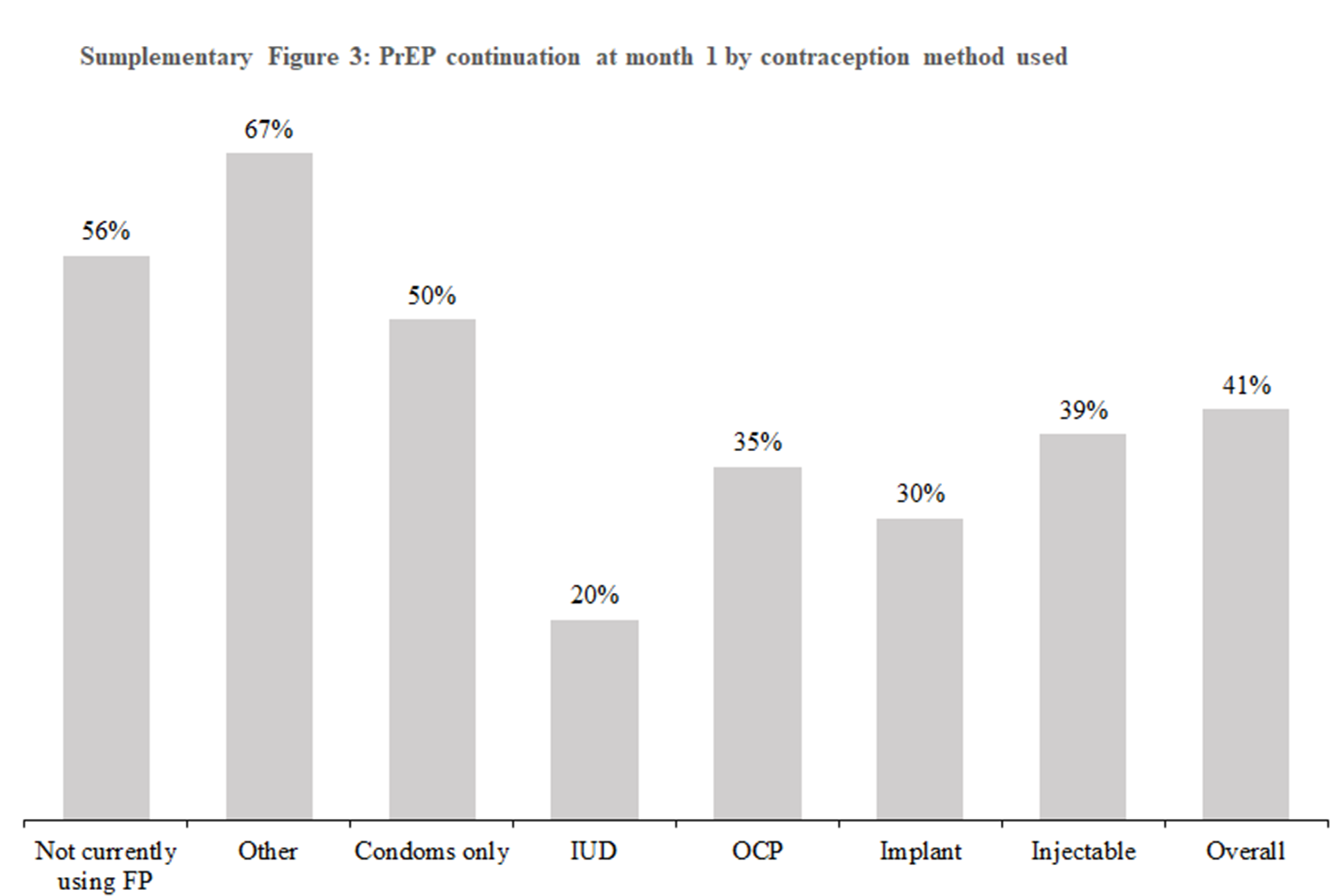

Supplement: Supplementary file 3 [file Image_3.TIF]
